# Heat resistance and presence of genes encoding staphylococcal enterotoxins evaluated by multiplex-PCR of *Staphylococcus aureus* isolated from pasteurized camel milk

**DOI:** 10.1042/BSR20191225

**Published:** 2019-11-13

**Authors:** Hany M. Yehia, Elsayed A. Ismail, Zeinab K. Hassan, Abdulrahman H. Al-masoud, Mosffer M. Al-Dagal

**Affiliations:** 1 Food Science and Nutrition Department, College of Food and Agricultural Sciences, King Saud University, Riyadh, Saudi Arabia; 2 Food Science and Nutrition Department, Faculty of Home Economics, Helwan University, Cairo, Egypt; 3 Department of Dairy Science, Faculty of Agriculture, Benha University, Benha 13518, Egypt; 4 Cancer Biology Department, Virology and Immunology Unit, National Cancer Institute, Cairo University

**Keywords:** Staphylococcus aureus, D-value, enterotoxin C, heat resistant, pasteurized camel milk

## Abstract

Milk pasteurization eliminates vegetative pathogenic microorganisms and reduces microorganisms associated with spoilage. Camel milk is a well-accepted, traditionally consumed food in Arab countries. The present study aimed to investigate the microflora of pasteurized camel milk sold in Riyadh City, Saudi Arabia. The heat resistance of the microflora was tested in culture medium and lab-sterilized milk, and its composition was verified by multiplex polymerase chain reaction (PCR) using specific primers. Further verification was performed by using separate specific primers. The identified strain survived heat treatment at 65, 72, 80, 85, and 90°C for 30, 15, 10, 5, and 2 min, respectively. An unanticipated result was obtained when an enterotoxin producing strain of *Staphylococcus aureus* showed abnormal resistance to heat treatment. The enterotoxin gene within the PCR fragment was identified as enterotoxin C by DNA sequencing. During Basic Local Alignment Search Tool (BLAST) analysis, the isolated enterotoxin C genes showed >99% similarity to published database sequences of the *Staphylococcus aureus* strain SAI48 staphylococcal enterotoxin C variant v4 (*sec*) gene**.** The decimal reduction value (D-value) at 90°C *(D_90_*) was determined after 10 s. This is the first time to report this abnormally heat resistant and enterotoxin-producing strain of *Staphylococcus aureus*. The use of ultra-high temperatures (UHTs) is preferable for reducing or killing bacteria in camel milk, especially if this problem is encountered in many camel milk factories.

## Introduction

Toxin producing strains of *Staphylococcus aureus* can cause diseases in humans and animals. Their pathogenicity is attributable to toxin production and the antibiotic-resistant nature of these strains [1]*.* Furthermore, *S. aureus* strains that are foodborne pathogens are referred to as toxin producing strains [2]. The enterotoxins produced by *S. aureus* (*SEs*) have been classified into five types based on serology, including *sea, seb, sec, sed*, and *see* [1]. Approximately 16 new types of *SEs* (*seg, seh, sei, ser, ses*, and *set*) and *SELS* (*selj, selk, sell, selm, seln, selo, selp, selq, selu*, and *selv*) have been described [1].

The genes for staphylococcal enterotoxins A (*sea*) and E (*see*) are carried by a temperate bacteriophage [3–5]. Enterotoxins B (*seb*) and C (*sec*), as well as toxic shock syndrome toxin 1 (TSST-1), are located on the chromosome [5,6], while enterotoxins D (*sed*) and J (*sej*) are carried by plasmids [5].

In general, milk is a good medium for the growth of *S. aureus* and the production of their enterotoxins. Staphylococcal toxins are very resistant to heat [7]. Enterotoxins retain some of their biological activity in milk after pasteurization or heating at 121°C for 28 min [8]. Camel milk is considered to be one of the most important types of milk consumed in Saudi Arabia and several other Arab countries. The advantage of using the camel over other species is its ability to provide high quantity milk. In addition, camel milk has many medicinal characteristics and is consumed by people with anemia, food allergies, spleen diseases, asthma, jaundice, piles, and dropsy [9]. It is important to care for the health of camels and to improve their breed to increase their production of milk and meat. Though camel milk is thought to provide immunity against most of the diseases that usually affect other livestock, it has been found to contain a high microbial load, which threatens the lives of calves [10].

Camel milk has been found to be contaminated with several bacterial species that caused mastitis, such as *Staphylococcus, Streptococcus, Escherichia coli, Pseudomonas, Corynebacterium*, and *Mycobacterium tuberculosis*, as well as fungal species such as *Aspergillus, Candida*, and *Cryptococcus* [11,12]. All these organisms can invade the teat and mammary glands, where the bacteria can proliferate and produce toxins, which affects milk secretion from the tissues and leads to an increased somatic cell count. It was found that raw and pasteurized camel milk can be contaminated by *S. aureus* [8], which is introduced from different parts of the animal’s body or through the method of handling, various processing steps, or unhygienic environmental conditions.

Camel milk is normally pasteurized at 63°C for 30 min or 72°C for 15 s. *S. aureus* was isolated from milk treated in this way; the presence of this organism was not affected by pasteurization and remained active, which has led to the thought that these strains may be more heat resistant. These strains of *S. aureus*, known to exhibit heat resistance and produce toxin C (*sec*), are thought to be more dangerous and harmful for human health compared with other toxin-producing strains that can be inactivated during the normal pasteurization process [13].

The aim of this work was to study an *S. aureus* strain isolated from pasteurized camel milk sold in the local markets of Riyadh, S.A. The isolates were studied with regard to (a) heat resistance at different temperatures and (b) the presence of genes coding for *sec* by using multiplex-polymerase chain reaction (PCR).

## Materials and methods

### Camel milk samples

Fifty pasteurized camel milk samples were purchased from local markets in Riyadh (S.A.) and analyzed by different biochemical and genetic identification tests.

### Bacterial strain

*S. aureus* ATCC 29737 and *S. epidermidis* ATCC 12228 were used as the reference strain and negative control strain, respectively. *S. aureus* was isolated from pasteurized camel milk on (Oxoid CM0275) supplemented with egg yolk tellurite emulsion (SR0054). The agar plates were incubated at 37°C for 48–72 h. Characteristic colonies were tested for catalase and coagulase production using rabbit plasma (bioMérieux; Lyophilized Rabbit Plasma, Ref. 55182) and by evaluating blood haemolysis. Five presumptive *S. aureus* colonies were then tested following API STAPH-IDENT strip system techniques.

### API STAPH-IDENT strip system

The API STAPH tests (bioMérieux) were performed according to the manufacturer’s instructions. Identification is carried out using the numerical profiles of the positive samples within each group; a 7-digit profile number is obtained using databases (V4.1) and the analytical profile index.

### Phenotypic assessment of slime-synthesizing *S. aureus* strains using Congo red agar

The slime produced by the standard *S. aureus* ATCC 29737 strain and the *Staphylococcus* isolates was assessed quantitatively based on the amount of colour developed on Congo red agar. The agar was prepared by adding the following components to 1 litre of distilled water: Tryptone Soya Broth (TSB, Oxoid CM0129); 30 g), sucrose (36 g), agar powder (20 g), and Congo red (0.8 g) [14]. *S. aureus* ATCC 29737, *S. epidermidis* ATCC 12228, and the *Staphylococcus* isolates were struck on to Congo red agar and incubated for 24 h at 37°C under aerobic conditions. The isolates were compared with the standard *S. aureus* ATCC 29737 (slime producer) strain, which was considered as a positive control, and *S. epidermidis* ATCC 12228 (non-slime producer) was considered as a negative control. Slime production results were interpreted as follows: strains producing intensive black, black, and reddish black colonies with a rough, dry, or crystalline consistency were considered to be normal slime producers, whereas those producing smooth red and Bordeaux red-coloured colonies were classified as non-slime producers, as reported previously [15].

### Detection of Staphylococcal enterotoxin genes by PCR test

Total genomic DNA was isolated from the *S. aureus* isolates by using a commercial kit (AxyPrep™ Bacterial Genomic DNA Miniprep Kit, Cat. no. AP-MN-BT-GDNA 50; Axygen Scientific, Inc., U.S.A.) according to the supplier’s instructions. Table 1 shows the primers that were used for the detection of the *SEs* genes. The PCR was carried out using 10 µl of 5× FIREPol® Master Mix Ready to Load (with 12.5 mM MgCl_2_; Solis BioDyne, Tartu, Estonia), 2 µl of primer mix (diluted from a 100 µM stock of each primer), 5 µl of DNA template, and 33 µl of ultrapure water. The DNA amplification was performed in a MultiGene Thermal Cycler (Labnet International, Inc., Edison, NJ) under the following conditions: initial denaturation was carried out for 5 min at 95°C, followed by 30 cycles of denaturation (94°C for 1 min), annealing (50°C for 1 min), elongation (72°C for 1 min), and a final elongation (72°C for 10 min). A final extension step (72°C for 5 min) was performed after the completion of the cycle. Aliquots of the PCR products along with a 1 kb DNA ladder (GeneCraft, GC-015-003) were loaded into a 1.5% agarose gel (Sigma–Aldrich, Cat. no. 9539) containing Ethidium Bromide (0.5 mg/ml; ROTH, Cat. no., 12182) and electrophoresis was carried out using 10× TBE buffer (Tris-Borate-EDTA; BIO BASIC INC., Cat. no. A0026) for 30 min at 100 V (Electrophoresis Power Supply Model Consort EV243, Belgium). The amplified DNA fragments were visualized using visual image analyser (SYNGENE) software.

**Table 1:** Primer design and temperatures used for the detection of *S. aureus* enterotoxin encoding genes

Gene	Primer	Sequence (5′-3′)	Base pairs	Annealing temperature (°C)	Reference
SEA	SEA F	TTGGAAACGGTTAAAACGAA	120	50	Johnson et al*.* (1991)
	SEA R	GAACCTTCCCATCAAAAACA			
SEB	SEB F	TCGCATCAAACTGACAAACG	478	50	Johnson et al. (1991)
	SEB R	GCAGGTACTCTATAAGTGCC			
SEC	SEC F	GACATAAAAGCTAGGAATTT	257	50	Johnson et al. (1991)
	SEC R	AAATCGGATTAACATTATCC			
SED	SED F	CTAGTTTGGTAATATCTCCT	317	50	Johnson et al. (1991)
	SED R	TAATGCTATATCTTATAGGG			
SEE	SEE F	AGGTTTTTTCACAGGTCATCC	209	50	Mehrotra et al. (2000)
	SEE R	CTTTTTTTTCTTCGGTCAATC			

Abbreviations:** SEA**, *S. aureus* enterotoxin **A**; **SEB**, *S. aureus* enterotoxin **B**; **SEC**, *S. aureus* enterotoxin **C**; **SED**, *S. aureus* enterotoxin **D**; **SEE**, *S. aureus* enterotoxin **E**.

### Identification of the *sec* genotype by direct DNA sequencing

To identify integration and genotypes of the *S. aureus* enterotoxin C genes, DNA direct DNA sequencing was carried out on the PCR positive products. The PCR products were purified using a purification kit (Axygen® AxyPrep™ PCR Clean-Up) according to the manufacturer’s instructions (Corning, NY 14831, U.S.A.). According to Al-Shabanah et al. (2013) [16], the purified PCR products were labelled with fluorescent dyes using the BigDye Terminator v3.1 Cycle Sequencing Kit from Applied Biosystems. Labelled oligonucleotides were purified using the BigDye X Terminator Purification Kit (Applied Biosystems, CA, U.S.A.). The samples were sequenced by an automatic ABI 3500 genetic analyser (Applied Biosystems, U.S.A.). Chromatograms with sharp peaks, quality values ≥ 20, and little or no background noise were considered to belong to a single enterotoxin C gene. The nucleotide sequence was compared with the GenBank database using the Basic Local Alignment Search Tool (BLAST, provided by the National Cancer Institute, U.S.A.) (http://blast.ncbi.nlm.nih.gov/Blast.cgi).

### Antimicrobial susceptibility testing

Freshly carried out antimicrobial susceptibility tests were compared between the standard *S. aureus* ATCC 29737 strain and the *S. aureus* enterotoxin C-producing strain. The tested bacterium was obtained from an overnight culture (inoculated from a single colony) in brain heart infusion broth (BHI; Oxoid, CM1135), which was plated on to Mueller-Hinton agar (Oxoid, CM405) using the agar disk diffusion method (CLSI, 2018) [17]. A total of 19 antibiotic discs (Oxoid, U.K.) containing the following components were prepared and tested against the bacteria: 75 μg ticarcillin (TIC 75), 25 μg colistin sulphate (CT 25), cefoxitin (FOX), 30 μg cefadroxil (CFR 30), 25 μg sulphamethoxazole trimethoprim (SXT 25), 25 μg ampicillin (AML 25), 5 μg cloxacillin (OB 5), 30 μg linezolid (LZD 30), 30 μg tetracycline (TE 30), 5 μg ciprofloxacin (CIP 5), 30 μg vancomycin (VA 30), 300 μg Nitrofurantoin (F300), 10 μg colistin sulphate (CT) 15 μg erythromycin (E 15), 30 μg amoxicillin (AML 25), 30 μg Cephalothin (KF 30), 10 μg Bacitracin (B), PB = 300 U Polymyxin B (PB), and 30 µg chloramphenicol (C 30). Diameters of the zones of inhibition (mm) were interpreted using the criteria recommended for *S. aureus* by NCCLS (2018) [18]. Based on the diameters of the antibiotic inhibitory zones, the isolates were classified as either sensitive (S) or resistant (R) strains. The results were interpreted based on the sizes of the inhibition zones. Zones of inhibition were considered to be sensitive, intermediate, or resistant (NCCLS, 2018) [18].

### *D*-value determination

The *D*-value, or decimal reduction value, is known as the time required to inactivate 90%, or 1 log, of the initial population at a given temperature. Solutions containing enterotoxin C-producing *S. aureus* and the standard *S. aureus* ATCC 29737 strain (control) were prepared for thermal destruction or inactivation trials. Active cultures were obtained by inoculating one colony of the *S. aureus* strains into BHI broth (Oxoid, CM1135) and allowing them to grow at 37°C for 24 h. One millilitre of active 24 h culture was diluted into screw-top test tubes containing 9 ml of BHI medium (active cultures of *S. aureus* tend to clump; hence, the cells must be vortexed at maximum speed for 1 min prior to dilution in BHI medium). The tubes with diluted solutions (in duplicate) were exposed to heat in a water bath at 90°C for 0, 10, 20, 30, 40, 50, and 60 s. After each heat treatment, the tube was quickly cooled down in an ice water bath for 20 s. For the enumeration of the surviving cells, serial dilutions were performed, and 1 ml of each sample was poured on to BHI agar and incubated at 37°C for 24–48 h. The colony forming units per ml (CFU/ml) were recorded for every treatment. The optical densities of the diluted tubes were recorded after incubating at 37°C for 24 h. The D-values were determined from the linear section of the survivor plots using linear regression analysis. D-values are reported in seconds and are defined as the time required to achieve a 1 log reduction in the bacterial population at a designated temperature. Before heat treatment, the number of cells in the inoculated medium ranged from 10^6^ to 10^7^ per ml.

### Z-value determination

The *Z-*value is the temperature increase required to decrease the number of organisms by 1 log at a specified *D-*value. The Z-values were determined at different temperatures – 65, 70, 75, 80, 85, 90, and 95°C – and compared with the *D*-values; the *Z*-values were calculated using the formula Z = slope − 1 (the temperature change necessary to induce a 10-fold change in the *D*-value) [19].

## Results

The results, provided in Table 2, showed that the *Staphylococcus* isolates were negative for catalase and positive for coagulase, while *S. aureus* ATCC 29737 was positive for both catalase and coagulase. Colonies of the *Staphylococcus* isolates were surrounded by clear zones on sheep blood agar, similar to that observed for the β-haemolysis of the reference *S. aureus* ATCC 29737 strain. After the mannitol was fermented in mannitol coagulase agar, the pH of the medium surrounding the coagulase positive colonies changed, and the Bromocresol Purple indicator turned yellow, presenting yellow zones around the colonies. An opaque area composed of coagulated plasma was found around the colonies of coagulase positive organisms. *S. aureus* ATCC 29737 and the *Staphylococcus* isolates fermented the mannitol in mannitol salt agar to produce yellow coloured colonies surrounded by yellow zones. Positive DNase activity on DNase agar was visualized as clear zones around the colonies when the plates were flooded with 1 N hydrochloric acid. The results, summarized in Table 2, showed that the *Staphylococcus* isolates and the *S. aureus* ATCC 29737 strains were similar. The *S. aureus* ATCC 29737 strain and the *S. aureus* isolates were compared and analyzed by assays performed using the API STAPH system.

**Table 2:** Phenotypic characterization based on laboratory tests using the API STAPH system for the detection and identification of the *S. aureus* isolates

	Tests						API STAPH system tests																				
Microorganisms	*Catalase	*Growth on Columbia agar + 5% blood sheep haemolysis	*Growth on mannitol salt agar	*Growth on mannitol coagulase agar	*Growth on DNase agar	*Coagulase	Glucose	Fructose	Mannose	Maltose	Lactose	Trehalose	Mannitol	Xylitol	Melibiose	B-naphthyl phosphate	Potassium nitrate	Sodium pyruvate	Raffinose	Xylose	Sucrose	Methyl α D-glucopyranoside-D-glucopyranoside	N-acetyl glucosamine	l-arginine	Urea	7-digit numerical profile by API STAPH	Identification ratio (%)
*S. aureus* ATCC 29737	+	+	+	+	+	+	**+**	+	−	**+**	**+**	**+**	**+**	−	−	**+**	**+**	**+**	−	−	**+**	−	**+**	**+**	−	6 7 3 6 1 5 3	***S. aureus***
*Staphylococcus* isolates	−	+	+	+	+	+	**+**	**+**	**+**	**+**	**+**	**+**	+	−	+	−	**+**	**+**	−	−	−	−	**+**	**+**	−	6 7 3 5 1 4 1	***S. aureus***

* Tests not included in the API STAPH system.

### Detection of *Staphylococcus* enterotoxin genes by PCR

**Figure 1:**
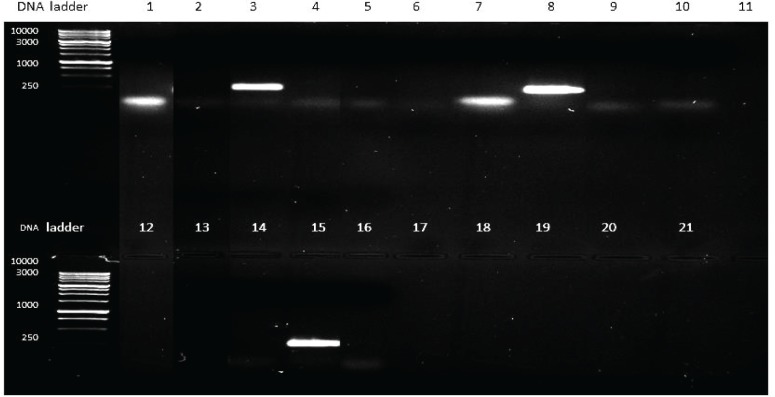
PCR product generated by using genomic DNA multiplex primers for *S. aureus* toxins *S. aureus* ATCC 29737 (sea, lanes 1 and 7; approximately 120 bp) and Staphylococcal isolates (**lanes 2**–**21**). Positive toxin C bands appeared in isolates 3, 8, and 15 and were approximately 257 bp in length, as visualized after gel electrophoresis using 1% agarose and 10 µl Ethidium Bromide with an image analyser (SYNGENE).

**Figure 2:**
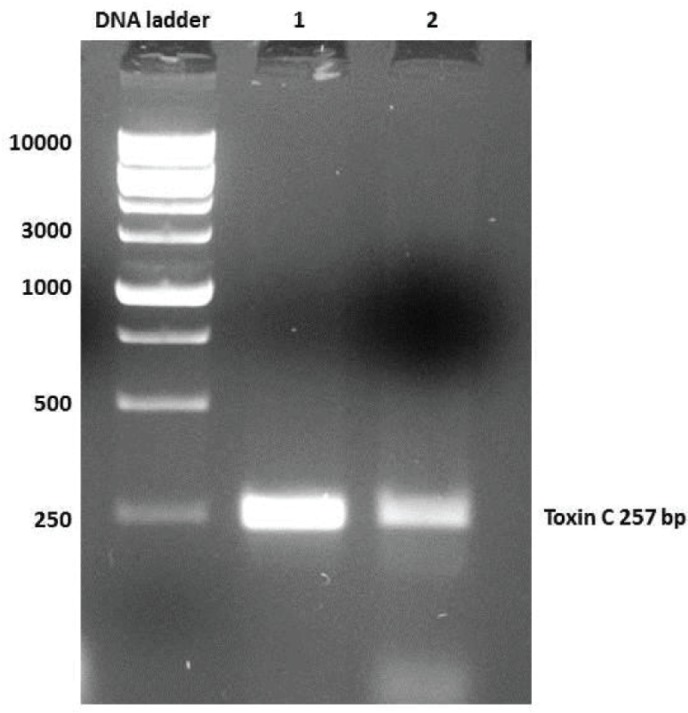
PCR product Lanes (1 and 2) generated by using specific *S. aureus* toxin C primers (257 bp).

For the detection of toxin genes in the *Staphylococcus* strains, genomic DNA was extracted from all 21 isolates obtained from camel milk and from the *S. aureus* ATCC 29737 control strain. PCR was run using multiplex toxin gene primers (A, B, C, D, and E). As shown in Figure 1, the band size observed in lanes 3, 8 and 15 corresponded to a *sec* gene of approximately 257 bp; the *S. aureus* ATCC 29737 *sea* gene was used as a positive control (lane 1). To verify that the bands that appeared were specific to toxin C, another PCR was run by using only toxin C primers (forward and reverse) and the PCR products from the three positive isolates (isolates 3, 4, and 8) under the same conditions in a thermocycler*.* The results, shown in Figure 2, confirm that the PCR products were enterotoxin C because they were 257 bp in length.

### Genotyping by direct DNA sequencing

Direct DNA sequencing of the *S. aureus* PCR amplicon using the *sec* F primer as the forward sequencing primer was used to obtain the unique hypervariable regions of the *S. aureus* strain. The submission of this 225-bp sequence to GenBank for BLAST alignment analysis returned a report that indicated that this sequence was located in a chromosomal region from position 619 to 672, which confirmed that this sequence had a unique 99% ID match with the sequence of the staphylococcal enterotoxin C variant v3 (*sec*) gene in *S. aureus* strain SAI3 (sequence ID: KX168614.1).

Similarly, by using the *sec* R primer as the reverse sequencing primer, it was possible to obtain the unique hypervariable regions downstream of the *sec* F primer binding site in the *S. aureus* strain. The submission of this 229-bp sequence to GenBank for BLAST alignment analysis returned a report that indicated that this sequence was located in the chromosomal region between 595 and 782. This result confirmed that this sequence had a unique 99% ID match with the sequence of the staphylococcal enterotoxin C variant v3 (*sec*) gene in *S. aureus* strain SAI3 (Sequence ID: KX168615.1).

**Figure 3:**
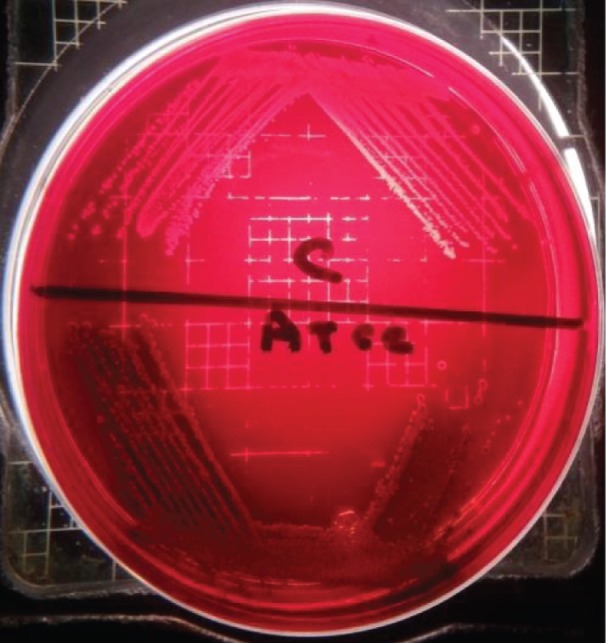
Slime productions on Cong agar medium *S. aureus* ATCC 29737 (ATCC) the medium turned to black, and a lack of slime production and black colony by the *S. aureus* enterotoxin C-producing strain (C).

As shown in Figure 3, slime production was not detected in the *S. aureus* enterotoxin C-producing strain, in contrast with the *S. aureus* ATCC 29737 strain. Although the *S. aureus* enterotoxin C-producing strain produced toxin C and was coagulase positive and blood haemolytic (β-haemolytic), we speculated that slime production may not be associated with the infectivity of this strain.

### Antimicrobial susceptibility test

Table 3 demonstrates the effect of antibiotics, which were tested against the *S. aureus* enterotoxin C-producing isolates; the effects were similar to those against *S. aureus* ATCC 29737. These results indicate that the *S. aureus* enterotoxin C-producing strain was appropriately identified by all tests and was detected by PCR; further, the strain may be sensitive to TIC 75, FOX 30, CFR 30, SXT 25, AMP 10, LZD 30, CIP 5, E 15, AMC 30 and C 30, resistant to CT 25, OB 5, TE 30, CT 10, B 10, PB 300, and intermediately sensitive to CIP 5, VA 300 and KF 30.

**Table 3:** Antimicrobial disk susceptibility tests for *S. aureus* ATCC 29737 and the *S. aureus* enterotoxin C-producing strain on Mueller-Hinton

Strain	Antibiotics																		
	TIC 75 ND	CT 25 ND	FOX 30 21 - 21	CFR 30 ND	SXT 25 16 11-15 10	AMP 10 15 - 14	OB 5 ND	LZD 30 23 21-22 20	TE 30 19 15-18 14	CIP 5 21 16-20 15	CT 10 ND	VA 30 17 16-15 14	F 300 17 16-15 14	E 15 23 14-22 13	AMC 30 20 - 19	KF 30 ND	B 10 ND	PB 300 ND	C 30 18 13-17 12
*Staphylococcus aureus*	35	15	22	20	25	30	12	26	12	20	7	16	15	25	25	15	12	8	20
ATCC 29737	S	R	S	S	S	S	R	S	R	I	R	R	I	S	S	I	R	R	S
*Staphylococcus aureus* enterotoxin	35	12	25	20	20	35	15	25	12	20	7	15	17	25	30	15	12	10	20
C-producing strain	S	R	S	S	S	S	R	S	R	I	R	I	I	S	S	I	R	R	S

Mean zones of inhibition for common antibiotics tested: S = Sensitive, I = Intermediate, R = Resistant, ND = Not detected. TIC 75 = Ticarcillin (75 μg), CT 25 = Colistin (25 μg), FOX 30 = Cefoxitin (30 μg), CFR30 = Cefadroxil (30 μg), SXT 25 = Sulphamethoxazole trimethoprim (25 μg), AMP 10 = Ampicillin (10 μg), OB 5 = Cloxacillin (5 μg), LZD 30 = Linezolid (30 μg), TE 30 = Tetracycline (30 μg), CIP 5 = Ciprofloxacin (5 μg), CT = Colistin (10 and 25 μg), VA 30 = Vancomycin (30 μg), F 300 = Nitrofurantoin (300 μg), E 15 = Erythromycin (15 μg), AMC = Amoxicillin/Clavulanic Acid (30 μg), KF 30 = Cephalothin (30 μg), B = Bacitracin (10 μg), PB = Polymyxin B (300 U), C 30 = Chloramphenicol (30 μg).

### Thermal inactivation

**Figure 4:**
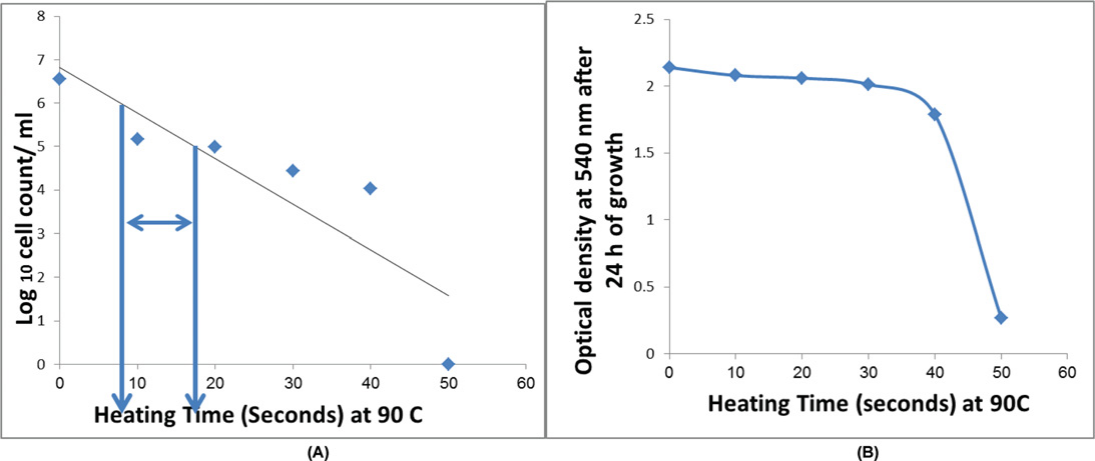
Effect of heating tempaeratures on growth rate of S. aureus enterotoxin C producing strain (**Left**) Survivor curve showing a Log decrease in the number of *S. aureus* enterotoxin C-producing strain at 90°C. Where *D_90_*°C is number of unit time it takes for the survivor curve to traverse 1 log 10 unit cycle. (**Right**) Optical density of *S. aureus* enterotoxin C-producing strain. Decrease in the growth was noted after the 24-h heat treatment at 90°C for 0, 10, 20, and 30 min.

The *S. aureus* enterotoxin C-producing strain and *S. aureus* ATCC 29737 were heat treated first at 65°C for 30 min, then at 72°C for 15 min, and finally at 85°C for 10 min. After heat treatment, the strains were streaked on to BHI agar, and the plates were incubated at 37°C for 48–72 h. Bacterial growth was noted after all three temperature treatments for both *S. aureus* ATCC 29737 and the *S. aureus* enterotoxin C-producing strain. As a result, the temperature treatments were increased to 90°C for 0, 10, 20, 30, 40, 50, and 60 s to determine the D-value of the *S. aureus* enterotoxin C-producing strain, as shown in Figure 4 (Left). We found that the *D_90_* °C value ranged from 8 to 10 s. Additionally, the optical density was checked to determine the growth of the surviving bacteria in BHI broth after 24 h of incubation at 37°C, as shown in Figure 4 (Right). The growth was reduced by increasing the time of exposure to heat at 90°C; the OD values were 2.2, 2.1, 2.0, 2.0, 1.8, and 0.2 after 0, 10, 20, 30, 40, and 50 s, respectively.

**Figure 5:**
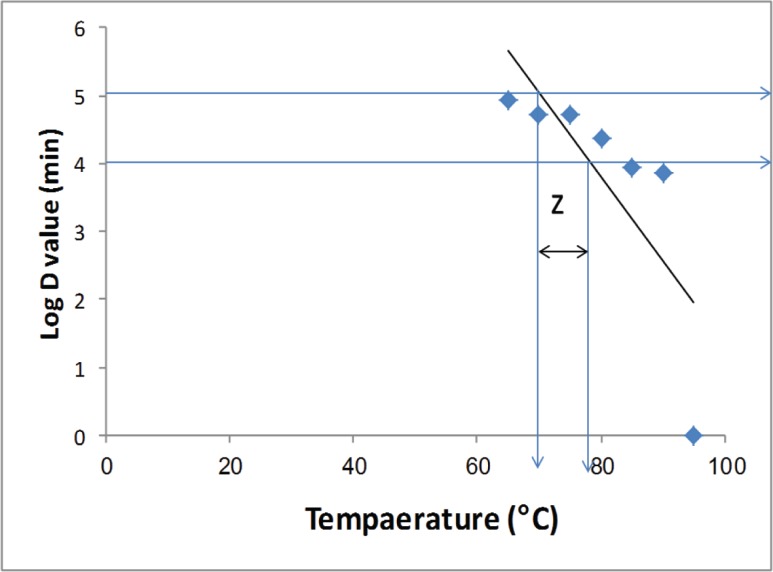
Thermal death time curves for the *S. aureus* enterotoxin C-producing strain Estimated based on D-values determined in BHI broth medium at different temperatures.

The Z-value for the *S. aureus* enterotoxin C-producing strain ranged from 8 to 10 s at 65, 70, 75, 80, 85, 90, and 95°C; thus, 10 s would be required to achieve a 1 log reduction at 90°C, as shown in Figure 5. For the most resistant strains, the *Z*-values are approximately 10 s, which has also been adopted as a standard *Z-*value.

## Discussion

Coagulase production was considered evidence that our isolates were toxin-producing organisms. These results corresponded to those obtained by Di Salvo (1958) [20], who confirmed that there is a correlation between coagulase activity and DNase activity because of the incorporation of DNA into the medium along with calcium chloride, which activates the enzyme. Our isolate was identified as *S. aureus*, and its identification ratio was 97% according to the Apiweb™ identification software.

There are many virulence factors that increase the potential of disease development by *S. aureus*, some of which are described as toxins secreted by *S. aureus* that interfere directly with the host (Otto, 2014) [21]. Other authors, such as Christensen et al. (1982) [22], Davenport et al. (1986) [23], Kleeman et al. (1993) [24], Ammendolia et al. (1999) [25] and Mack et al. (2000) [26], mentioned that this virulence factor is associated with the production of slime by some strains of *Staphylococcus*. Slime production has been found in coagulase-negative *Staphylococcus* (CNS) in the form of a loosely bound exopolysaccharide capsule (slime layer), which has been associated with sepsis, including intravenous catheter-related bacteraemia and other prosthetic device infections [27–29]. Similarly, it was found that some strains of *S. aureus* contained bacterial capsules, which are closely associated with the bacterial cell wall. These strains may also contain an extracapsular and labile extrapolysaccharide structure [30].

*S. aureus* ATCC 29737 was found to contain a *sea* gene of approximately 120 bp, and these data agreed with Han et al. (2016) [31].

Slime production by *S. aureus* has been thought to be virulence factor. Recently, some authors reported a higher colonization capacity of *S. aureus* strains producing slime compared with their non-slime producing counterparts. Hence, slime producing *S. aureus* strains might play a role in the establishment of infection (Ammendolia et al. (1999)) [25]. Some authors declared that the resistance to high concentrations of antibiotics might be greater in slime producing *S. aureus* strains compared with their non-slime producing counterparts [32].

No difference was found between the two strains of *S. aureus*, which indicates that their behaviour towards antibiotics is not related to their enterotoxin genes but may be due to physiological or cytological changes. When used for the growth of injured *S. aureus* cells, liquid or solidified enrichment media, such as BHI agar, was more efficient than other complex or specific media, such as Baird-Parker agar, which might lead to a delay in growth. Similar milk containing components that are more nutritive would be conducive to the growth of *S. aureus* as the dominant microbe after heat treatment by pasteurization. *S. aureus* ATCC 29737 did not grow after any exposure time at 85°C or 90°C. The exposure time varies considerably among different strains of microorganisms and might be similar between the same genus and species, depending on the treatment utilized and the food contaminated. The heating environment and treatment of foods always differ according to the nature of their components. Heat pasteurization (63°C and 72°C for 30 min and 15 s, respectively) has always been used in the treatment of milk, while other heat treatments, such as boiling vegetables and fruits at 100°C or boiling acidified foods for sterilization at 121°C for 15–20 min, have also been used for sterilizing canned meat products and canned legumes. Additionally, exposing the same microorganism to different heat treatments might lead to the appearance of heat-resistant strains of *S. aureus*, such as the strain of *S. aureus* that was isolated from pasteurized camel milk in our study; hence, this brings our attention to the fact that the microbial contamination may have occurred after milk pasteurization, from the milk packaging, or during the pasteurization process, which was not efficient enough to destroy all contaminating milk microorganisms. The authors previously determined the D-values of *S. aureus* in prechilled storage conditions and in storage conditions without chilling and found that the *D_50_, D_55_*, and *D_60_* values ranged from 94.3 to 127.9 min, 13 to 21.7 min, and 4.8 to 6.5 min, respectively. The *Z*-value is defined as the temperature change necessary to bring about a ten-fold change in the *D*-value [33]. Kennedy et al. (2005) [33] found that Z-values of *S. aureus* ranged from 7.7 to 8.0 min at temperatures of 61, 62, 63, 64, 65, 66, 67, 68, 69, 70, 71, and 72°C. These results suggested, for example, that approximately 24 s would be required to achieve a 1 log reduction at 70°C.

In recent years, more attention has been paid to camel milk because it contains casein hydrolysates with high antioxidant activity, which can be used in the development of functional foods [34]. As a result, there has been a focus on thermal processing treatments that result in high quality milk free from contaminated or heat-resistant bacteria, while at the same time preserving its components. Camel milk is also used in different countries, and for example, it is used in many products in India; in India, thermally concentrated milk is used to make khoa, and it has been found that khoa prepared from camel milk has the highest moisture and ash contents and lower fat, protein and lactose contents compared with khoa prepared from cow and buffalo milk samples. The acidity, soluble nitrogen, free fatty acids, and peroxide values of the khoa prepared from camel milk were found to be higher than those found in khoa prepared from cow and buffalo milk [35]. In Saudi Arabia, fermented camel milk has shown potential health benefits and has antimicrobial effects against microorganisms such as *Bacillus cereus, Salmonella typhimurium*, and *S. aureus*, whereas unfermented camel milk exhibited no antimicrobial effects against any of the tested pathogens [36].

Some authors have suggested the possibility of using pulsed UV-light treatment as an alternative method for complete inactivation of *S. aureus* in milk, which can be potentially adapted to a commercial setting for continuous milk pasteurization [37]. Others have suggested that the combined effects of inoculating milk with some of the tested bacteriocin-producing lactic acid bacteria (BP-LAB) and high-pressure treatments (HPT) of cheese were synergistic on *S. aureus* inactivation [38]. The combination of high-pressure processing and temperature change can reduce the amount of *S. aureus* by approximately 5 log units at 4°C and approximately 8 log units at 50°C [39].

We can conclude that if the camel milk pasteurization process does not include increased use of disinfectants or sanitizers with continuous cleaning, then some strains of *S. aureus* with the highest heat resistance may grow on the pipes and trays used for milk treatment. The discovery and dispersal of a toxin-producing strain of *S. aureus* that is resistant to heat treatment at 90°C poses a high risk to human health because most people who consume this kind of milk do not treat it with any additional heat, and the symptoms of food poisoning usually develop within 30 min to 8 h. The combination of mild heat treatments and high-pressure treatments may be alternative solutions to this problem.

## Abbreviations

BHI: Brain heart infusion
BLAST: Basic Local Alignment Search Tool
D-value: Decimal reduction value
PCR: Polymerase chain reaction
SE: Enterotoxins produced by *Staphylococcus aureus*

## References

[1] Argudin M.A., Mendoza M.C.and Rodicio M.R. (2010) Food poisoning and *Staphylococcus aureus* enterotoxins. Toxins 2, 1751–1773 10.3390/toxins2071751 22069659 PMC3153270

[2] Hennekinne J.A., Ostyn A., Guillier F., Herbin S., Prufer A.L.and Dragacci S. (2010) How should Staphylococcal food poisoning outbreaks be characterized. Toxins 2, 2106–2116 10.3390/toxins2082106 22069675 PMC3153283

[3] Betley M.J.and Mekalanos J.J. (1985) Staphylococcal enterotoxin A is encoded by phage. Science 229, 185–187 10.1126/science.3160112 3160112

[4] Borst D.W.and Betley M.J. (1994) Phage-associated differences in staphylococcal enterotoxin A gene (sea) expression correlate with sea allele class. Infect. Immun. 62, 113–118 8262616 10.1128/iai.62.1.113-118.1994 PMC186075

[5] Zhang S., Iandolo J.J.and Stewart G.C. (1998) The enterotoxin D plasmid of *Staphylococcus aureus* encodes a second enterotoxin determinant (*sej*). FEMS Microbiol. Lett. 168, 227–233 10.1111/j.1574-6968.1998.tb13278.x 9835033

[6] Novick R.P., Schlievert P.and Ruzin A. (2001) Pathogenicity and resistance islands of Staphylococci. Microbes Infect. 3, 585–594 10.1016/S1286-4579(01)01414-9 11418332

[7] Anderson J.E., Beelman R.B.and Doores S. (1996) Persistence of serological and biological activities of Staphylococcal enterotoxin A in canned mushrooms. J. Food Protect. 59, 1292–9 10.4315/0362-028X-59.12.1292 31195505

[8] Rall V.L.M., Vieira F.P., Rall R., Vieitis R.L., Fernandes A. Jr, Candeias J.M.G. et al. (2008) PCR detection of staphylococcal enterotoxin genes in *S. aureus* strains isolated from raw and pasteurized milk. Vet. Microbiol. 132, 408–413 10.1016/j.vetmic.2008.05.011 18572331

[9] Viguier C., Arora S., Gilmartin N., Welbeck K.and O’Kennedy R. (2009) Mastitis detection: current trends and future perspectives. Trends Biotechnol. 27, 486–493 10.1016/j.tibtech.2009.05.004 19616330

[10] Al-Ruwaili M.A., Khalil O.M.and Selim S.A. (2012) Viral and bacterial infections associated with camel (*Camelus dromedarius*) calf diarrhea in North Province, Saudi Arabia. Saudi J. Biol. Sci. 19, 35–41 10.1016/j.sjbs.2011.10.001 23961160 PMC3730540

[11] Khan M.and Khan A. (2006) Basic facts of mastitis in dairy animals: a review. Pak. Vet. J. 26, 204–208

[12] Al-Majali A., Bani Ismail Z., Al-Hami Y.and Nour A. (2007) Lactoferrin concentration in milk from camels (*Camelus dromedarius*) with and without subclinical mastitis. Int. J. Appl. Res. Vet. Med. 5, 120–124

[13] Fung D.Y.C., Steinberg D.H., Miller R.D., Kurantnick M.J.and Murphy T.F. (1973) Thermal Inactivation of *Staphylococcal* Enterotoxins B and C. Appl. Microbiol. 26, 938–942 4767298 10.1128/am.26.6.938-942.1973 PMC379937

[14] Freeman D.J., Falkiner F.R.and Keane C.T. (1989) New method for detecting slime production by coagulase negative Staphylococci. J. Clin. Pathol. 42, 872–874 10.1136/jcp.42.8.872 2475530 PMC1142068

[15] Kouidhi B., Zmantar T., Hentati H.and Bakhrouf A. (2010) Cell surface hydrophobicity, biofilm formation, adhesives properties and molecular detection of adhesions genes in *Staphylococcus aureus* associated to dental caries. Microb. Pathog. 49, 14–22 10.1016/j.micpath.2010.03.007 20298773

[16] Al-Shabanah O.A., Hafez M.M., Hassan Z.K., Sayed-Ahmed M.M., Abozeed W.N., Al-Rejaie S.S. et al. (2013) Human papillomavirus genotyping and integration in ovarian cancer Saudi patients. Virol J. 10, 343 24252426 10.1186/1743-422X-10-343 PMC3842654

[17] Clinical and Laboratory Standards Institute: CLSI Guidelines (2018) M-100 Performance Standards for Antimicrobial Susceptibility Testing, 28th edn., Clinical and Laboratory Standards Institute, Wayne, PA

[18] NCCLS (National Committee for Clinical Laboratory Standards) (2002) Performance Standards for Antimicrobial Susceptibility Testing: Twelfth Informational Supplement NCCLS Document M100-S12, NCCLS, Wayne, PA, U.S.A.

[19] Holdsworth S.D. (1997) Thermal Processing of Packaged Foods, pp. 70–111, Blackie Academic and Professional, London

[21] Di Salvo J.W. (1958) Desoxyribonuclease and coagulase activity of Micrococci. Med. Technicians Bull. 9, 191–196 13577185

[21] Otto M. (2014) *Staphylococcus aureus* toxins. Curr. Opin. Microbiol. 0, 32–37 10.1016/j.mib.2013.11.004 PMC3942668 24581690

[22] Christensen G.D., Simpson W.A., Bisno A.L.and Beachey E.H. (1982) Adherence of slime producing strains of Staphylococcus *epidermidis* to smooth surfaces. Infect. Immun. 37, 318–326 6179880 10.1128/iai.37.1.318-326.1982 PMC347529

[23] Davenport D.S., Massanari R.M., Pfaller M.A., Bale M.J., Streed S.A.and Hierholzer W.J. (1986) Usefulness of a test for slime production as a marker for clinically significant infections with coagulase negative *Staphylococci*. J. Infect. Dis. 153, 332–339 10.1093/infdis/153.2.332 2935582

[24] Kleeman K.T., Bannerman T.L.and Kloos W.E. (1993) Species distribution of coagulase-negative Staphylococcal isolates at a community hospital and implications for selection of *Staphylococcal* identification procedures. J. Clin. Microbiol. 31, 1318–1321 8501235 10.1128/jcm.31.5.1318-1321.1993 PMC262927

[25] Ammendolia M.G., Di Rosa R., Montanaro R., Arciola C.R.and Baldassarri L. (1999) Slime production and expression of the slime-associated antigen by staphylococal clinical isolates. J. Clin. Microbiol. 37, 3235–3238 10488184 10.1128/jcm.37.10.3235-3238.1999 PMC85536

[26] Mack K.D., Bartscht S., Dobinsky M.A., Horskotte H., Kiel K.M., Knobloch A. et al. (2000) Staphylococcal factors involved in adhesion and biofilm formation on biometarials. In Handbook of Bacterial Adhesion. Principles Methods and Applications(An Y.H.and Friedman R.J., eds), pp. 307–330, Humana Press, Totowa, NJ

[27] Ishak M.A., Groschel D.H., Mandell G.L.and Wenzel R.P. (1985) Association of slime with pathogenicity of coagulase-negative *Staphylococci* causing nosocomial septicemia. J. Clin. Microbiol. 22, 1025–1029 4066912 10.1128/jcm.22.6.1025-1029.1985 PMC271871

[28] Diaz-Mitoma F., Harding G.K.M., Hoban D.J., Roberts R.S.and Low D.E. (1987) Clinical significance of a test for slime production in ventriculoperitoneal shunt infections caused by coagulasenegative Staphylococci. J. Infect. Dis. 156, 555–560 10.1093/infdis/156.4.555 3624905

[29] Etienne J., Brun Y., Solh N.E., Delorme V., Mouren C., Bes M. et al. (1988) Characterization of clinically significant isolates of *Staphylococcus epidermidis* from patients with endocardatis. J. Clin. Microbiol. 26, 613–617 3366858 10.1128/jcm.26.4.613-617.1988 PMC266386

[30] Caputy G.G.and Costerton J.W. (1982) Morphological examination of the glycocalyses of *Staphylococcus aureus* strains Wiley and Smith. Infect. Immun. 36, 759–767 7085077 10.1128/iai.36.2.759-767.1982 PMC351295

[31] Han J.I., Yang C.H.and Park H.M. (2016) Prevalence and risk factors of *Staphylococcus spp*. carriage among dogs and their owners: a cross-sectional study. Vet. J. 212, 15–21 10.1016/j.tvjl.2015.10.059 27256020

[32] Kloss W.E.and Bannerman T.L. (1994) Update on clinical significance of coagulase-negative *Staphylococci*. Clin. Microbiol. Rev. 7, 117–140 10.1128/CMR.7.1.117 8118787 PMC358308

[33] Kennedy J., Blair I.S., McDowell D.A.and Bolton D.J. (2005) An investigation of the thermal inactivation of *Staphylococcus aureus* and the potential for increased thermotolerance as a result of chilled storage. J. Appl. Microbiol 99, 1229–1235 10.1111/j.1365-2672.2005.02697.x 16238754

[34] Fayza M.A., Mona A.M.A., Jihan M.K.and Mohamed H.A. (2018) Proteolysis and antioxidant activity of peptic, trypticand chymotryptic hydrolysates of cow, buffalo, goatand camel caseins. In J. Dairy Technol. 71, 236–242 10.1111/1471-0307.12400

[35] Milankumar J.C., Bhavbhuti M.M., Devesh H.P., Vijaykumar B.D.and Kishorkumar D. (2016) Characterisation and comparison of khoa prepared fromcamel milk with that from cow and buffalo milk. Int. J. Dairy Technol. 2, 253–260

[36] Omar A.A., Ali A.M., Esayed A.I., Hatem S.A., Abdulrahman S.A.and Ara D.K. (2018) Angiotensin converting enzyme-inhibitory activity andantimicrobial effect of fermented camel milk (Camelus dromedarius). Int. J. Dairy Technol. 70, 27–35

[37] Krishnamurthy K., Demirci A., and Andirudayaraj J.M. (2007) Inactivation of *Staphylococcus aureus* in milk using flow-through pulsed UV-light treatment system. J. Food Sci. 72, M233–9 10.1111/j.1750-3841.2007.00438.x 17995646

[38] Arques J.L., Rodriguez E., Gaya P., Medina M., Guamis B.and Nunez M. (2005) Inactivation of *Staphylococcus aureus* in raw milk cheese bycombinations of high-pressure treatments and bacteriocin-producing lactic acid bacteria. J. Appl. Microbiol. 98, 254–260 10.1111/j.1365-2672.2004.02507.x 15659179

[39] Windyga B., Rutkowska M., Sokołowska B., Skąpska S., Wesołowska A., Wilińska M. et al. (2015) Inactivation of *Staphylococcus aureus* and native microflora in human milk by high pressure processing. High Press Res. 35, 181–188 10.1080/08957959.2015.1007972

